# Comparison of Treatment Effects of Different Iron Chelators in Experimental Models of Sepsis

**DOI:** 10.3390/life11010057

**Published:** 2021-01-14

**Authors:** Christian Lehmann, Maral Aali, Juan Zhou, Bruce Holbein

**Affiliations:** 1 Department of Anesthesia, Pain Management and Perioperative Medicine, Dalhousie University, Halifax, NS B3H 4R2, Canada; juan.zhou@dal.ca; 2 Department of Physiology and Biophysics, Dalhousie University, Halifax, NS B3H 4R2, Canada; M.Aali@dal.ca; 3 Department of Microbiology and Immunology, Dalhousie University, Halifax, NS B3H 4R2, Canada; bholbein@cpintercept.com

**Keywords:** iron, iron chelation, inflammation, infection, sepsis

## Abstract

Growing evidence indicates that dysregulated iron metabolism with altered and excess iron availability in some body compartments plays a significant role in the course of infection and sepsis in humans. Given that all bacterial pathogens require iron for growth, that iron withdrawal is a normal component of innate host defenses and that bacterial pathogens have acquired increasing levels of antibiotic resistance, targeting infection and sepsis through use of appropriate iron chelators has potential to provide new therapeutics. We have directly compared the effects of three Food and Drug Administration (FDA)-approved chelators (deferoxamine—DFO; deferiprone—DFP; and deferasirox—DFX), as were developed for treating hematological iron overload conditions, to DIBI, a novel purpose-designed, anti-infective and anti-inflammatory water-soluble hydroxypyridinone containing iron-selective copolymers. Two murine sepsis models, endotoxemia and polymicrobial abdominal sepsis, were utilized to help differentiate anti-inflammatory versus anti-infective activities of the chelators. Leukocyte adhesion, as measured by intravital microscopy, was observed in both models, with DIBI providing the most effective reduction and DFX the poorest. Inflammation in the abdominal sepsis model, assessed by cytokine measurements, indicated exacerbation by DFX and DFO for plasma Interleukin (IL)-6 and reductions to near-control levels for DIBI and DFP. Peritoneal infection burden was reduced 10-fold by DIBI while DFX and DFP provided no reductions. Overall, the results, together with those from other studies, revealed serious limitations for each of the three hematological chelators, i.e., as potentially repurposed for treating infection/sepsis. In contrast, DIBI provided therapeutic benefits, consistent with various in vitro and in vivo results from other studies, supporting the potential for its use in treating sepsis.

## 1. Introduction

Iron is an essential element for most forms of life, as its redox-cycling capabilities enable its participation in numerous biochemical reactions [1]. However, iron levels must be tightly regulated to prevent excess availability to invading microbes and avoid free iron toxicity via catalysis of reactive oxygen species (ROS) [2]. Inadequately regulated iron levels and excessive ROS production have been linked to the dysregulated immune response in sepsis [3]. At present, there are no pharmacological therapies approved for the treatment of immune dysregulation in clinical sepsis. Additionally, antibiotic resistance is increasing, making it even more difficult to treat severe infections such as sepsis [4]. Iron chelators are potentially capable of addressing both needs by restricting iron availability for ROS production and reducing bacterial growth through nutritional limitation.

Iron chelators have been introduced clinically to treat patients with iron overload conditions such as hemochromatosis and in thalassemia, where a defect in hemoglobin production reduces erythropoiesis and, thus, frequent blood transfusions are needed, causing increased iron levels. Currently, three Food and Drug Administration (FDA)-approved iron chelators are available: deferasirox (DFX), deferoxamine (DFO) and deferiprone (DFP). These are small molecules that can enter host cells to lower excess intracellular iron stores.

Deferasirox (Exjade^®^) is a tridentate iron chelator and was the first oral medication approved for chronic iron overload induced by long-term blood transfusion. Two molecules of DFX bind to a single molecule of Fe^3+^ to create an intracellular complex [5]. It has a long half-life of 8–16 h, which provides the advantage of less frequent dosing, resulting in increased patient compliance with treatments [6].

Deferoxamine (Desferal^®^) is a tri-hydroxamate with the ability to bind both extracellular aluminum and iron. A single molecule of DFO binds to an Fe^3+^ iron and forms a stable complex. Due to its low lipophilicity, it cannot be administered orally and is instead administered intramuscularly (if patients are not in shock) and, slowly, intravascularly (if patients are in shock or experiencing cardiovascular collapse) [7]. It has a bi-phasic half-life of a 1-h rapid phase followed by a 6-h slow phase and it is eliminated renally [8]. DFO is a microbial siderophore derived from *Streptomyces pilosus* and is thus associated with the risk of supplying the iron to bacteria during infection.

Deferiprone (Ferriprox^®^) is an orally administered bidentate iron chelator. Three molecules of DFP are needed to chelate a single molecule of iron. It has a half-life of 2–3 h and is excreted through urine. Although it can bind to other metals (zinc, copper and aluminum), it has a higher affinity for iron.

DIBI is a water-soluble linear co-polymer functionalized with nine 3-hydroxypyridin-4-one chelating moieties that act cooperatively to more selectively and strongly bind Fe^3+^ [9]. Unlike the above three hematological chelators, DIBI was designed as an antimicrobial, anti-inflammatory agent, and compared to DFP, DIBI has over 1000× higher antimicrobial activity. Additionally, DIBI has a higher formation constant (Log ß_3_ for iron complexation) than the other chelators with a measured Log ß_3_ = 41.05 compared to DFP with Log ß_3_ = 36.7 [9].

We sought to investigate the anti-inflammatory and anti-bacterial effects of the three FDA-approved iron chelators and compared their efficacy to DIBI in two experimental models of sepsis (endotoxemia and colon ascendens stent peritonitis (CASP)-induced sepsis). For this, the four chelators were each administered at equimolar iron-binding capacities. Using intravital microscopy (IVM), a unique imaging modality to study the immune response in vivo, we assessed sepsis-induced leukocyte–endothelial interactions and capillary perfusion in the intestinal microvasculature. Additionally, we analyzed selected plasma cytokines (tumor necrosis factor (TNF)-α, Interferon (IFN)-γ, Interleukin (IL)-1β and IL-6) and bacterial infection burdens as well as changes in the peritoneal microbiome in experimental animals with CASP-induced sepsis. Overall, our findings suggest a promising role for iron chelation as a novel approach for attenuating the dysregulated inflammatory response and a potential anti-bacterial therapy for clinical sepsis.

## 2. Materials and Methods

### 2.1. Ethics Statement

The experimental procedures were approved by the University Committee on Laboratory Animals at Dalhousie University (protocols #17-070, #18-057 and #18-090) and performed in accordance with the guidelines and standards of the Canadian Council on Animal Care.

### 2.2. Animals

Wild-type male C57BL/6 mice (8–10 weeks old; 20–30 g) were purchased from Charles River Laboratories International Inc. (Saint-Constant, QC, Canada) and acclimated for one week in ventilated plastic cage racks in a pathogen-free room in the Carleton Animal Care Facility of the Faculty of Medicine, Dalhousie University, Halifax, NS, Canada. They were housed in a 12-h light/dark cycle at 21 °C with supply of a standard diet of rodent chow and filtered water ad libitum.

### 2.3. Endotoxemia Model

#### 2.3.1. Anesthesia and Surgery

Mice were anesthetized by intraperitoneal (IP) injection of sodium pentobarbital (90 mg/kg; Ceva Sainte Animale, Montreal, QC, Canada). Anesthesia depth was monitored every 15 min by pedal withdrawal reflex assessment. Additional sodium pentobarbital was given as needed (5 mg/kg IP). Mice were placed in supine position on a heating pad. Body temperature was measured continuously via a rectal thermometer connected to a feedback warming system (TCAT-2LV Controller; Physitemp Instruments, Clifton, NJ, USA) and body temperature was maintained at 37 °C.

The right anterior lateral neck area of the animal was shaved and disinfected. Next, a small incision was made in the right side of the neck to dissect the jugular vein. Using a silk thread, the cranial end of the vein was tied off. Using micro scissors, the vein was cut open and a catheter was inserted into the vein and pushed approximately 1 cm into the vessel. The catheter consisted of a 30-gauge needle inserted into a non-radiopaque polyethylene tubing (PE10, Clay Adams, Sparks, MD, USA) and was secured in place by knotting a silk thread around the catheter and both the inferior and superior ends of the vessel. The catheter was used for intravenous (IV) administration of the experimental substances.

#### 2.3.2. Experimental Timeline

Endotoxin (5 mg/kg lipopolysaccharide (LPS) from *Escherichia coli*; 1 mg/mL in normal saline (NS); Sigma-Aldrich, Oakville, ON, Canada) or vehicle (NS) was administered (IV) at T = 0. At T = 15 min, treatment (one of the iron chelators) or vehicle (NS) was administered IV. At T = 1 h 45 min, fluorochromes were injected IV. This was followed by laparotomy and intravital microscopy (IVM) of the intestine (T = 2 h).

#### 2.3.3. Experimental Groups

This model consisted of six experimental groups. Group 1, the control group, did not receive LPS. Instead, vehicle (NS) was administered. Groups 2–6 received LPS. In groups 3–6, we administered one of the iron chelators. To ensure that a similar iron-binding capacity was added, the dosages of the iron chelators were matched based on DIBI’s iron-binding capacity, i.e., assuming full hexadentate iron binding for each chelator. Group 3 was treated with DFX (2.25 mg/kg; Santa Cruz Biotechnology Inc., Dallas, TX, USA), group 4 received DFP (1.25 mg/kg; Sigma Aldrich, St. Louis, MI, USA) and group 5 was administered DFO (1.97 mg/kg; Sigma Aldrich, St. Louis, MI, USA). Group 6 received treatment with DIBI (10 mg/kg; provided by Chelation Partners Inc., Halifax, NS, Canada).

### 2.4. CASP-Induced Sepsis

#### 2.4.1. Anesthesia and Surgery

Anesthesia was induced by 4% isoflurane in oxygen (flowrate: 1 L/h) in an induction chamber. Depth of anesthesia was maintained by continuous inhalation of 2% isoflurane in oxygen (flowrate: 0.8 L/h) through a nose cone. Buprenorphine (0.1 mg/kg) was administered subcutaneously (SC) for post-operative pain relief and eye gel was applied.

CASP surgery was conducted aseptically and in accordance with Dalhousie University guidelines. The abdominal region was shaved and cleaned with detergent (hibitane) and disinfectants (isopropyl alcohol 70% solution followed by povidone-iodine). Laparotomy (midline incision) was then performed, and the ascending colon was gently retracted and placed over a sterile gauze on the animal’s body. A 20-gauge 1-1/4 catheter (Jelcro, Smiths Medical, Kent, UK) was securely placed at a 45° angle from the wall of ascending colon (1 cm distal to the ileocecal valve) using a 7-0 polypropylene monofilament suture (3304H, Pronova, Ethicon by Johnson & Johnson, New Brunswick, NJ, USA). After removing the needle, the catheter tube was cut to leave 2–3 mm of the stent projecting outside of the ascending colon. Using wet cotton-tipped applicators and gently palpating the cecum, feces filled the catheter and fecal matter entered the peritoneal cavity. The stent was tightly sutured in the colon for continuous fecal matter leakage. Next, treatment or vehicle (saline) was instilled into the abdominal cavity (IP). Sham surgery for healthy control mice was performed under the same conditions, except for the catheter insertion into the ascending colon. In these animals, the catheter was sutured to the top of the outer layer of the ascending colon, with care taken to avoid perforation and leakage of any fecal matter into the abdominal cavity. The ascending colon was positioned back into the abdominal cavity and the muscle layer was sutured using 5/0 non-absorbable polypropylene monofilament (7740G, Prolene, Ethicon by Johnson & Johnson, New Brunswick, NJ, USA). All mice were given 0.5 mL NS for fluid replacement (SC) and kept in a recovery area on a heating pad (37 °C) with rodent chow mash and water ad libitum.

#### 2.4.2. Experimental Timeline

The experiment began with anesthesia and CASP surgery (T = −45 min). Administration of treatment/vehicle at the end of the surgery marked the T = 0 timepoint. At T = 6 h 45 min, the mouse was anesthetized again. At T = 7 h 30 min (which marked 30 min prior to IVM), fluorochromes were administered IV through tail-vein injection. Re-laparotomy was also performed at this time along the prior CASP surgical incision. At T = 7 h 45 min, peritoneal lavage fluid (PLF) was collected for bacterial enumeration and microbiome analysis. IVM started at T = 8 h. Once IVM was completed, at T = 8 h 45 min, blood samples were collected and stored appropriately for further analysis.

#### 2.4.3. Experimental Groups

Six experimental groups were established: group 1 (sham) served as the surgical control group. Sham animals underwent the same surgery, except for the perforation of the ascending colon (absence of fecal matter leakage). At T = 0, sham animals received 0.2 mL/100 g body weight normal saline (NS) vehicle IP as treatment. The remaining five groups had active fecal matter leakage into the abdominal cavity. Group 2 (CASP) represents septic mice without treatment. Similar to sham animals, they received NS only at T = 0. Groups 3 to 6 received one of the iron chelators in NS IP. To ensure similar iron chelation capacity, the dosages of the iron chelators were again matched based on DIBI’s iron-binding capacity. Groups 3, 4 and 5 received DFX (18 mg/kg), DFP (10 mg/kg) and DFO (15.76 mg/kg), respectively. Group 6 received DIBI treatment (80 mg/kg).

### 2.5. Intravital Microscopy

#### 2.5.1. Preparation before Microscopy

Fluorochromes were administered IV 15 min before the scheduled IVM time. Rhodamine-6G (0.05%, 1.5 mL/kg; Sigma-Aldrich, Oakville, ON, Canada) was used to stain mitochondria and facilitate visualization of leukocytes. Fluorescein isothiocyanate–bovine serum albumin (FITC-BSA; 5%, 1 mL/kg; Sigma Aldrich, Oakville, ON, Canada) was administered to visualize the microvasculature.

Laparotomy was performed by midline incision. Once the abdominal cavity was exposed, a 2 cm loop of terminal ileum was placed on the microscopic stage with the animal in lateral position. The exposed part of the intestine was superfused with warm NS through the entire procedure. Mouse body temperature was maintained by placing the IVM stage over a heating pad.

#### 2.5.2. Microscopy

IVM was performed by using an epifluorescence microscope (Leica DMLM, Wetzlar, Germany) and a light source (LEJ EBQ 100; Carl Zeiss, Jena, Germany). Three anatomical structures were studied in the exposed intestinal section: venules in the submucosal layer, capillaries in the muscle layer and capillaries in the mucosal layer. The mucosal layer was exposed by using a cauterizer to gently open the intestine. For each anatomical site, six visual fields were selected randomly. Leukocyte–endothelial interactions (number of adhering leukocytes) in the venules and capillary perfusion in muscle layer and mucosal villi (functional capillary density) were analyzed. Visual fields were recorded for 30 s. Using Volocity software (Perkin Elmer, Waltham, MA, USA), the videos were captured using a digital EM-CCD camera C9100-02 with an AC-adapter A3472-07 (Hamamatsu, Herrsching, Germany). Once IVM was completed, mice were euthanized via cardiac puncture. Video analysis was performed offline, blinded, using ImageJ software (version 2.0.0-rc-69/1.52p, National Institute of Health, Bethesda, MD, USA).

#### 2.5.3. Offline Video Analysis

Adherent leukocytes in submucosal venules were defined as cells that did not move or detach from the endothelial lining within the observation period of 30 s; values are given as the number of cells per square millimeter of endothelial surface, calculated from the diameter and length of the vessel segment studied, assuming cylindrical geometry. For muscle layer analysis of capillary perfusion, the biggest rectangular cross-section in focus was selected as the region of interest. Functional capillary density (FCD) was calculated as follows: total length of all perfused vessels in the region of interest divided by the total area selected (cm/cm^2^). For the mucosal villi, 5–6 villi in focus were selected for FCD calculation.

### 2.6. Bacterial Counts

Blood and PLF samples were used for bacterial enumeration. PLF was collected after relaparotomy by instilling 2 mL of pre-warmed sterile saline into the abdominal cavity. The saline was gently mixed with the peritoneal fluid and content. On average, a 1.5-mL volume of PLF was obtained for bacterial enumeration and microbiome composition analysis. Blood samples were collected by cardiac puncture at the end of the experiments. Sterile phosphate buffered saline (PBS) was used to make serial dilutions (1:1 × 10 to 1:1 × 10^6^) that were spot-plated on tryptic soy agar plates (TSA; Millipore Sigma, Etobicoke, ON, Canada) using the triplicate spot method. The plates were counted after 16–24 h to determine the colony-forming units (CFUs) per mL (CFU/mL). Spots were only enumerated when distinct and clear colonies were observed (~30 colonies per spot). CFUs/mL were calculated by taking the mean value obtained from the triplicate spots and multiplying it by the dilution factor.

### 2.7. Microbiome Sequencing

PLF samples were used to study the microbiome of the abdominal cavity in CASP-induced sepsis. For microbiome profiling, the 16S Bacteria and Archaea Standard Operating Procedure was used, as described in detail by Comeau et al. [10]. In short, 16S gene fragments were extracted and quantified from samples by PCR. The samples were analyzed in duplicate using two sets of forward primers and two sets of reverse primers at 1:1 and 1:10 dilutions, alongside four negative PCR controls. The duplicate PCRs were then combined in one plate and visually verified by running an E-gel. The amplicons were cleaned up and normalized using a high-throughput Invitrogen SequelPrep 96-well plate kit. The samples, along with the negative control, were pooled to create a library. The library was then quantified by utilizing the Invitrogen Qubit double-stranded DNA high-sensitivity fluorescence-based method followed by Illumina MiSeq sequencing.

### 2.8. Plasma Inflammatory Mediators

To quantify the plasma levels of tumor necrosis factor (TNF)-α, Interleukin (IL)-1β, IL-6 and Interferon (IFN)-β, blood samples (1.0 mL) were drawn at the end of the CASP experiments using heparin-coated syringes. Blood samples were immediately centrifuged at 250× *g* for 10 min to obtain plasma. The plasma was stored at −80 °C until measurement. Cytokines and adhesion molecules were analyzed using the Procarta Multiplex Cytokine Assay kit from Affymetrix (Freemont, CA, USA) and a Bio-Plex instrument with Bio-Plex software (Bio-Rad, Mississauga, ON, Canada) according to the manufacturers’ instructions.

### 2.9. Statistical Analysis

Data were analyzed using GraphPad Prism 8 software (version 8.2.0 272; La Jolla, CA, USA). Normal distribution was confirmed using the Kolmogorov–Smirnov test. Data were analyzed using unpaired t-tests or a one-way ANOVA followed by the Newman–Keuls test for comparison of three or more groups. Data were expressed as mean value ± standard deviation (SD). Significance was assumed at *p* < 0.05.

## 3. Results

### 3.1. Endotoxemia Model

#### 3.1.1. Leukocyte–Endothelial Interactions

**Figure 1:**
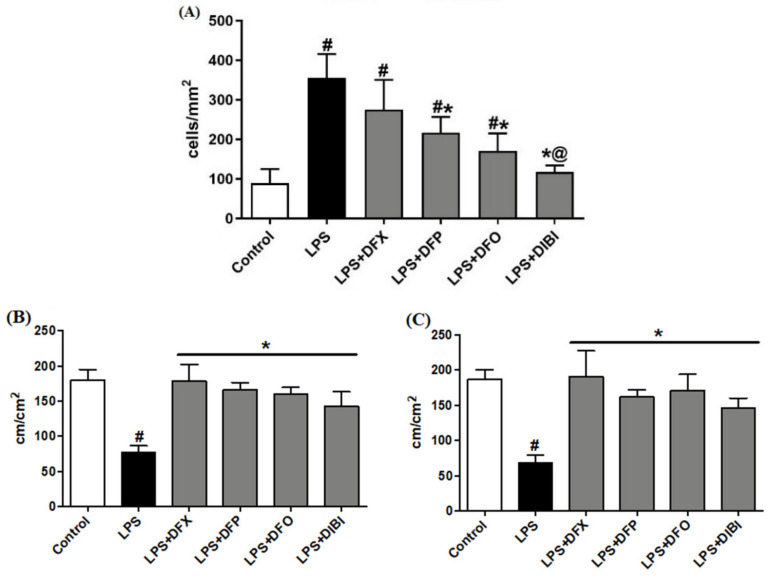
Iron chelation in endotoxemia—intravital microscopy. (**A**) Leukocyte adhesion in intestinal submucosal venules. Capillary perfusion in intestinal muscle layer (**B**) and mucosa (**C**). Data are represented as mean ± SD (*n* = 4–6 per group); ^#^ *p* < 0.05 compared to control group; * *p* < 0.05 compared to LPS group; @ *p* < 0.05 vs. deferasirox (DFX).

In the control group, we observed a low number of adhering leukocytes in submucosal venules (Figure 1). Mice challenged with LPS showed a substantial (approx. five-fold) increase in the number of adhering leukocytes, indicating the presence of systemic inflammation. Treatment with DFP, DFO or DIBI, respectively, significantly reduced LPS-induced leukocyte adhesion but DFX did not reduce adhesion significantly. DIBI treatment was the most effective, restoring leukocyte adhesion to near control levels.

#### 3.1.2. Capillary Perfusion

LPS-induced systemic inflammation significantly reduced FCD in muscle and mucosa layers when compared to the control group (Figure 1). All chelator treatments (DFX, DFP, DFO and DIBI) significantly improved the FCD to approximately control levels. There were no statistically significant differences in the FCDs among the various iron chelation treatment groups and healthy controls.

### 3.2. CASP-Induced Sepsis

#### 3.2.1. Leukocyte–Endothelial Interaction

**Figure 2:**
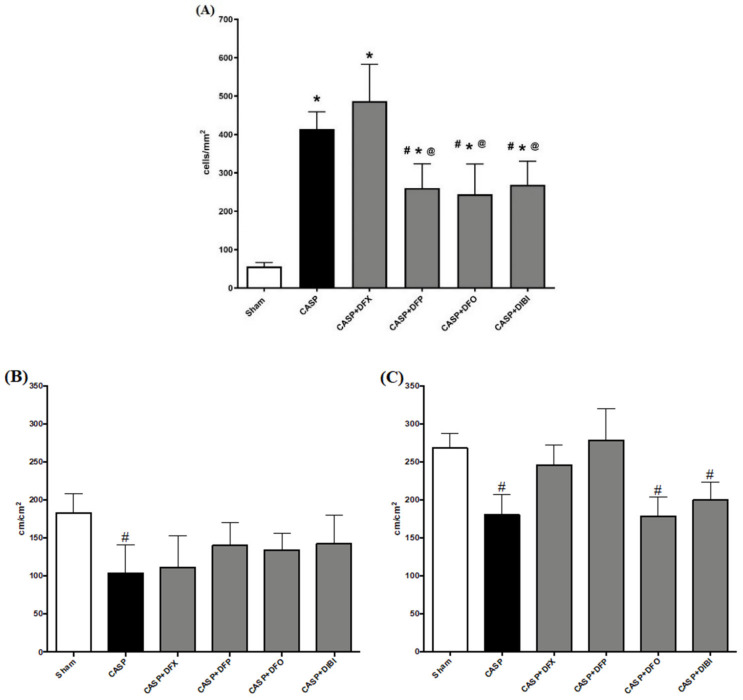
Iron chelation in colon ascendens stent peritonitis (CASP)-induced sepsis—intravital microscopy. (**A**) Leukocyte adhesion in intestinal submucosal venules. Capillary perfusion in intestinal muscle layer (**B**) and mucosa (**C**). Data are represented as mean ± SD (*n* = 4–6 per group); # *p* < 0.05 compared to control group; * *p* < 0.05 compared to LPS group; @ *p* < 0.05 vs. DFX.

Leukocyte adhesion in the submucosal intestinal venules of the sham group was at a low level (Figure 2). As expected, the number of adhering leukocytes significantly increased following the CASP procedure: all animals that underwent CASP surgery showed substantially increased (approx. eight-fold) numbers of adhering leukocytes compared to sham animals. Treatment with DFP, DFO or DIBI significantly lowered the number of adhering leukocytes compared to untreated CASP animals. DFX treatment was, again, not effective in reducing the number of adherent leukocytes in the infected mice.

#### 3.2.2. Capillary Perfusion

In both the muscle, and mucosal layers, FCD was significantly reduced in untreated CASP animals when compared to the sham group (Figure 2). In the muscle layer, we did not observe the same significant CASP-induced FCD reduction after treatment with the iron chelators. However, FCD levels in the iron chelation treatment groups were also not significantly different from untreated CASP animals. Within the mucosal layer, DFX and DFP treatments in CASP-induced sepsis improved the FCD substantially to the levels of sham animals.

#### 3.2.3. Plasma Inflammatory Mediators

**Figure 3:**
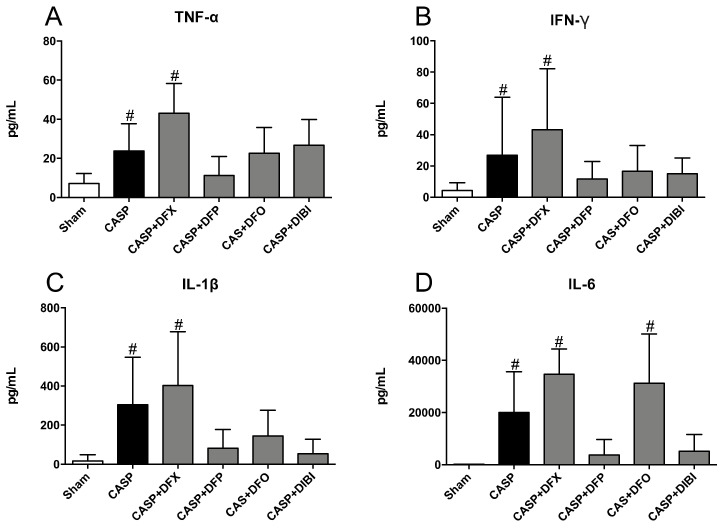
Iron chelation in colon ascendens stent peritonitis (CASP)-induced sepsis—plasma cytokines. (**A**) Tumor necrosis factor alpha (TNF-α), (**B**) Interferon gamma (IFN-γ), (**C**) Interleukin 1 beta (IL-1β), and (**D**) Interleukin 6 (IL-6) levels in plasma. Bar graphs represent mean cytokine values ± SD (*n* = 3–7); ^#^ *p* < 0.05 compared to control (sham) group.

TNF-α levels in sham CASP mice were very low (Figure 3A). After 8 h of infection, we measured significantly higher TNF-α levels in the untreated CASP animals and also CASP animals treated with DFX. TNF-α levels in CASP-infected animals treated with DFP, DFO or DIBI were not significantly elevated over sham controls.

IFN-γ levels increased significantly in untreated CASP animals and, again, in the DFX-treated CASP animals as compared to sham animals (Figure 3B). Treatment with DFP, DFO or DIBI reduced IFN-γ levels to near the level of sham animals.

IL-1β levels (Figure 3C) mimicked the pattern seen for TNF-α and IFN-γ; IL-1β was increased significantly in the untreated CASP group and the DFX-treated CASP group when compared to sham animals. The CASP groups treated with DFP, DFO or DIBI did not show significant increases in IL-1β levels compared to sham animals.

IL-6 levels were increased substantially in the untreated CASP group as well as in CASP animals treated with DFX or DFO compared to sham animals (Figure 3D). This CASP-induced IL-6 increase was not seen in CASP-infected animals treated with DFP or DIBI.

#### 3.2.4. Bacterial Counts

**Figure 4:**
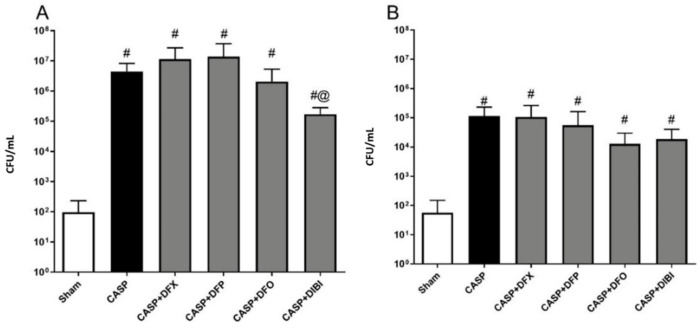
Iron chelation in colon ascendens stent peritonitis (CASP)-induced sepsis—bacterial enumeration. Bacterial burden was measured by plating peritoneal lavage fluid (PLF) samples (**A**) collected at 7 h 45 min and blood samples (**B**) collected at 8 h 45 min after CASP surgery on tryptic soy agar plates. The bacterial colonies were counted 16–24 h later (colony-forming units (CFU)/mL). Data are represented as mean ± SD (*n* = 4–8 per group); ^#^ *p* < 0.05 compared to sham group. @ *p* < 0.05 vs. CASP.

Bacterial burdens in both PLF (Figure 4A) and blood samples (Figure 4B) displayed similar patterns with bacterial counts being low in animals that underwent sham surgery. We saw significant bacterial burden increases following CASP surgery without iron chelation treatment. The bacterial counts were still significantly elevated in CASP animals treated with iron chelators as compared to sham animals, which was likely due to the ongoing fecal leakage. However, DIBI treatment significantly reduced (approx. 10× reduction) bacterial counts in PLF samples as compared to CASP-untreated mice.

#### 3.2.5. Microbiome Sequencing

**Figure 5:**
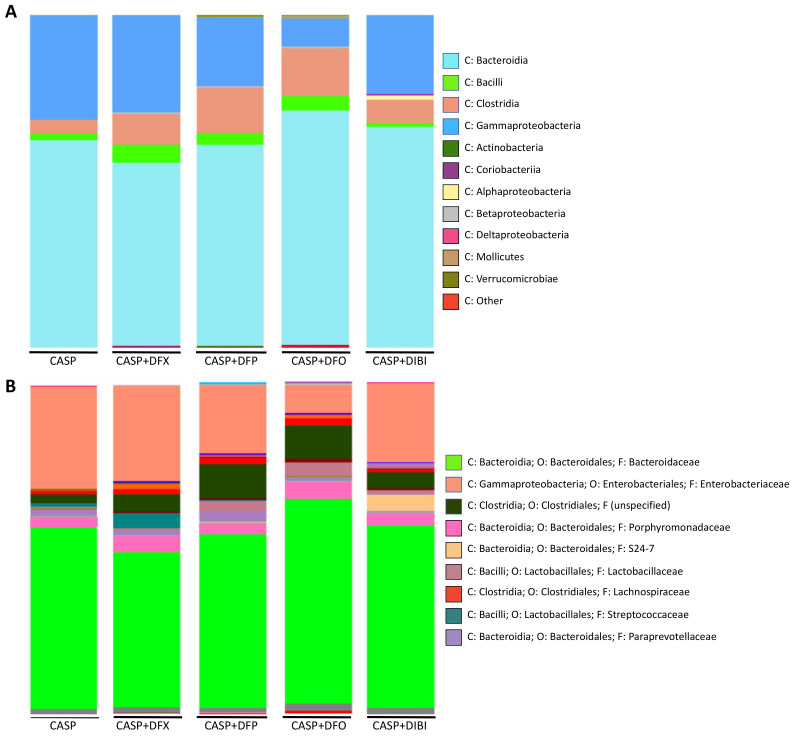
Iron chelation in CASP-induced sepsis—taxonomic composition of peritoneal lavage fluid microbiome at class and family levels. Grouped class (**A**) and family (**B**) bar graph plots of taxonomic composition of bacteria in peritoneal lavage fluid collected 7 h 45 min after CASP surgery.

The bacterial load in sham animals was too low for microbiome sequencing. In untreated CASP animals (Figure 5A), *Bacteroidia* was the dominant class of bacteria (62.2%), followed by *Gammaproteobacteria* (31.1%), *Clostridia* (4.3%) and *Bacilli* (1.8%), together forming the majority of the composition. These four classes were the most common classes within the various treatment groups as well, although their relative distributions displayed differences dependent on the particular iron chelator treatment.

A deeper analysis into the family level of CASP bacterial distribution revealed *Bacteroidaceae* (55%) and *Enterobacteriaceae* (31%) to be the most abundant members of the population of bacteria (Figure 5B).

Samples from DFX-treated mice were composed of *Bacteroidia* (54.9%), *Gammaproteobacteria* (29%), *Clostridia* (9.1%) and *Bacilli* (5.5%). The family distribution was similar to untreated CASP animals, with *Bacteroidaceae* (47%) and *Enterobacteriaceae* (29%) forming majority of the diversity. *Streptococcaceae* distribution was larger in DFX-treated animals.

Samples from DFP-treated mice contained *Bacteroidia* (60.3%), *Gammaproteobacteria* (20.7%), *Clostridia* (13.7%) and *Bacilli* (3.4%). In addition to the common *Bacteroidaceae* (52%) and *Enterobacteriaceae* (21%), DFP treatment appeared to have increased the distribution of bacteria from the *Clostridiales* (10%) order. The family of this bacterial order was not specified by the analysis library.

PLF samples from DFO-treated animals comprised *Bacteroidia* (70.4%), *Clostridia* (14.3%), *Gammaproteobacteria* (8.5%) and *Bacilli* (4.3%) classes. DFO-treated animals, interestingly, showed the lowest distribution of both *Gammaproteobacteria* class and *Enterobacteriaceae* family (8.5%). DFO treatment also resulted in the highest distribution of *Bacteroidia* (70.4%) and *Bacteroidaceae* (62%) compared to the other samples. DFO treatment also increased the relative population of *Clostridia* class (14.3%) significantly as compared to the CASP group. *Enterobacteriaceae* family (8.5%) of bacteria was found to have the smallest distribution within the DFO-treated animals as compared to all other groups.

Samples from DIBI-treated mice were composed of *Bacteroidia* (66.3%), *Gammaproteobacteria* (23.6%), *Clostridia* (7.0%) and *Bacilli* (1.1%) classes. Deeper family analysis revealed *Bacteroidaceae* (55%) and *Enterobacteriaceae* (24%) to be present. When compared to the other iron chelators, DIBI did not alter the *Bacilli* composition levels compared to CASP. It is important to note that the iron chelators, especially DFO, appeared to increase the *Clostridia* population relative to the other classes when compared to untreated CASP.

## 4. Discussion

### 4.1. Iron Chelation in Endotoxemia

The endotoxemia model enabled us to separate the impact of iron chelation on the immune response in sterile systemic inflammation from potential effects on bacterial growth. This model also has the advantage of administration of a reproducible amount of toxins in a controlled manner [11]. The selected DIBI dosage was chosen as it had demonstrated anti-inflammatory capacity in dose-ranging pilot experiments [12]. The other iron chelator dosages were correspondingly adjusted to provide similar iron-binding capacities to DIBI.

The pivotal part of the inflammatory response in sepsis is the recruitment of leukocytes to the site of inflammation [13]. Endothelial cells upregulate the expression of adhesion molecules to enable leukocyte–endothelial binding as the immune cells arrive through the blood stream to extravasate into the tissue [14]. The dynamic interactive process consists of several steps: rolling, adhering and emigration into tissue. Since firm adhesion is the critical step for consequent emigration, we defined leukocyte adhesion in submucosal venules as the primary endpoint of the study. The intestine is considered as a driving force in sepsis pathology [15] and is relatively easy to access surgically. Therefore, it was selected as the organ of interest for IVM.

Consistent with previous reports, LPS increased leukocyte adherence in intestinal submucosal venules [16,17,18]. LPS activates Toll-like receptor 4 (TLR4) and downstream inflammatory cascades that result in upregulation of adhesion molecule expression [19]. The DFP, DFO and DIBI treatments demonstrated systemic anti-inflammatory properties in the endotoxemia model of sepsis as they reduced the LPS-induced increase in the number of adhering leukocytes in the intestinal microcirculation. One possible explanation for this observation is the known antioxidative effect of iron chelation by blocking iron-related generation of reactive oxygen species (ROS) in the Fenton and Haber–Weiss reactions [20]. Consequently, ROS-dependent positive feedback mechanisms of leukocyte activation can be attenuated, and expression of adhesion molecules can be reduced. Though iron chelation treatments were matched for iron-binding capacity, administration of DFX or DIBI, respectively, showed significant contrasting differences in the impact on leukocyte adhesion. DFX has a weak metal-binding pyridinium group, which can hold iron in a redox-active state [21]. Consistent with this redox-reactive iron and our present findings, DFX has been reported to cause an increase in inflammatory factors and early renal damage in Thalassemic patients undergoing DFX therapy [22]. In contrast, DIBI holds iron in a redox-inactive state under physiological conditions [9]. These findings highlight the importance of the differing chemical and biological properties of the various iron chelators regarding their function.

Some effects of iron chelation can also be explained by considering other properties of these chelators aside from their iron-binding capacity such as their biological, pharmacological and chemical profiles. DIBI’s backbone is structurally similar to polyvinylpyrrolidone (PVP) and may have a plasma volume-expanding effect, acting like a colloid with anti-inflammatory effects [23,24,25,26]. Thus, DIBI’s active backbone may also make it more effective at modulating the immune response than through just exerting iron-chelation effects.

Reduction in leukocyte adhesion must be interpreted carefully, as a severe reduction (immune suppression) could be harmful if it impedes an adequate immune response and possibly impairs pathogen clearance; this highlights the need for a model with an active infection present.

### 4.2. Iron Chelation in CASP-Induced Sepsis

In our sham group, we observed a low-level baseline systemic leukocyte adhesion using intestinal IVM, most likely due to the surgical trauma (sham operation) that the mice underwent. Following the CASP procedure, we saw a significant rise in the number of adhering leukocytes during the infection, as we have previously shown [27]. In intestinal submucosal venules, DFP, DFO and DIBI reduced leukocyte adhesion compared to untreated CASP animals. This reduction was not a complete reversal to the level of sham animals. The goal of an effective iron chelation therapy in sepsis would be to reduce leukocyte hyperactivation yet still allow the immune system to generate an adequate response to the infection (i.e., pathogen clearance). Our results with DIBI are in agreement with previous findings. Leukocyte recruitment was reduced with DIBI alone or in combination with antibiotic co-treatment in the same CASP model of sepsis [27], and DIBI improved survival in CASP mice treated either with DIBI alone or especially when co-administered with imipenem [28]. In another model of sepsis infection using cecal ligation and puncture (CLP), therapy using DFO and the antioxidant N-acetylcysteine reduced oxidative stress and neutrophil infiltration and increased survival substantially [29]. Furthermore, in CLP-induced sepsis, DFO administration on its own improved survival in experimental animals [30].

In the plasma samples of sham animals, collected at 9 h post-sham surgery, inflammatory cytokine levels were low. CASP infection induced significant increases in all the measured cytokine levels. However, compared to systemic inflammation in the sterile endotoxemia model of sepsis, the cytokine levels were lower in CASP-induced sepsis. In endotoxemia, the peak of early pro-inflammatory cytokines such as TNF-α is reported to occur at 60 to 90 min post-LPS administration [31]. In the CASP model of sepsis, depending on the extent of fecal matter leakage, cytokine levels are expected to rise at 3 h and their peak has been shown to occur at 12 h after CASP surgery [32,33,34]. In the present study, blood samples were collected at 9 h post-CASP surgery, and therefore, lower cytokine levels were not unexpected. Furthermore, in endotoxemia models of sepsis, the inflammatory trigger, LPS, is often administered directly into the bloodstream, causing immediate and strong systemic inflammation. In CASP-induced sepsis, the infection is initially localized primarily in the abdominal cavity, and the systemic effects early during infection are therefore expected to be less pronounced compared to systemic endotoxemia. Nevertheless, iron chelation using DFP, DFO or DIBI, respectively, was able to reduce systemic cytokine release significantly, with DFP and DIBI being the most effective.

Interestingly, DFX administration did not modulate any of the cytokine levels, consistent with its inferior efficacy for reducing leukocyte adhesion as seen with IVM in both endotoxemia and CASP-induced sepsis cases.

As expected, we found high bacterial counts in the PLF and also the blood in CASP-induced sepsis. Because of the ongoing bacterial leakage induced by the CASP procedure with its continuous ingress of fecal bacteria, we were not able to measure the full quantitative anti-infective impacts of iron chelation therapy. However, there was an apparent trend of reduction in peritoneal bacterial burdens by both DFO and DIBI, with DIBI providing a significant 10× reduction in bacterial burden. In the same CASP model of sepsis, Islam et al. observed that the combination of DIBI and imipenem (beta-lactam antibiotic) reduced the bacterial burden significantly compared to the untreated group, but DIBI alone was not able to improve bacterial clearance [27]. However, that study assessed PLF and blood bacterial burdens after a full 16 h of infection, and there would have been substantially more fecal ingress to the peritoneal cavity.

Compared to the other iron chelators, DIBI has previously demonstrated superior anti-infective properties. In a study by Holbein and Orduna, DIBI effectively limited the growth of the opportunistic pathogens *Candida albicans* and *Candida vini* over a four-day period, while DFP and DFO did not [35]. DIBI has a high selectivity for both iron and manganese compared to other transition metals and is transparent to calcium and magnesium [35]. The antimicrobial effect of DIBI was reversed upon iron supplementation, emphasizing DIBI’s iron-specific effects. Moreover, DIBI (but not DFP or DFO) enhanced the efficacy of azoles (anti-fungals) in reducing the growth of clinical isolates of *C. albicans* [36]. DIBI also demonstrated synergistic effects with fluconazole to lower vaginal fungal burden, but alone, it did not reduce fungal burden [36].

The gut microbiome is a dynamic microbial consortium and is constantly responding and adapting to environmental changes and host factors. During the early stage of poly-bacterial abdominal infection induced by leakage of fecal matter from the gut, aerobic bacteria such as *E. coli* would replicate in and colonize the abdominal cavity. However, microcirculatory disruptions observed in sepsis can progressively create an anaerobic environment where obligate anaerobes such as *Bacteroidaceae* can replicate and lead to secondary infection, as has been observed in the chronic stage of infection [37]. As iron is a requirement for pathogenic bacteria, we expected that iron deprivation (via iron chelation) might affect changes in the species diversity of the peritoneal infection microbiome. Thus, the microbiome composition of the bacteria in PLF samples was analyzed. Low environmental iron creates competition, leaving some species susceptible and others more resistant to these changes. Though there was variability within the groups, we observed some interesting overarching trends.

The *Bacteroidaceae* family from *Bacteroidia* class formed the majority of the microbial composition in our PLF samples. *Bacteroidia* are Gram-negative, obligate anaerobic bacteria that are part of the normal gut microbiome. Though they are normally non-pathogenic commensals, if the intestinal barrier is compromised, such as through gastrointestinal rupture or intestinal surgery, they can translocate to the peritoneal cavity and the blood stream or surrounding tissues. In this family, *Bacteroides fragilis* is an opportunistic aerotolerant pathogen that can cause infections within the peritoneal cavity and is considered the leading anaerobic bacterium in sepsis and bacteremia [38]. Species from the *Bacteroidaceae* family are also known to have the highest rates of antibiotic resistance of all anaerobic pathogens [37]. Thus, with the rise in antibiotic-resistant strains (for example, to beta-lactams and aminoglycosides), exploiting their need for iron can represent a therapeutic target. During extra-intestinal infections, *Bacteroidaceae* acquire iron from heme and inorganic sources, although the exact mechanisms are under investigation [39]. We did not observe microbiome changes in this class. However, in a CASP experiment with a second dose of DIBI administered IV, *Bacteroidia* were almost entirely eliminated [12]. Perhaps *Bacteroidia* were more susceptible to iron deprivation from DIBI when growing in the bloodstream versus the abdominal cavity, as was assessed in the present study.

The second most common family of bacteria found in the PLF samples was the *Enterobacteriaceae* from the *Gammaproteobacteria* class. This large and diverse Gram-negative family includes highly virulent opportunistic human pathogens that release potent immune-stimulatory toxins. This family includes *Escherichia*, *Salmonella*, *Acinetobacter*, *Pseudomonas*, *Shigella*, *Vibrio*, *Yersinia* and other genera, with the most common for abdominal bacterial sepsis being *E. coli*, *P. aeruginosa* and *Klebsiella* spp., all of which have evolved complex iron-acquisition systems [40]. Interestingly, DFO-treated animals had the lowest proportion of *Gammaproteobacteria* class and *Enterobacteriaceae* family. This is in contrast to what has been reported before. As iron contributes to virulence and pathogenicity, DFO use in patients with Beta thalassemia is associated with higher risk for *Y. enterocolitica* septicemia [41]. *Y. enterocolitica* and other genera utilize iron bound to siderophores (DFO is one such siderophore) for growth in vitro, whereas DFP did not promote *Y. enterocolitica* growth, most likely due to DFP’s higher affinity for iron and non-microbial origin, making it harder for *Y. enterocolitica* to access the bound iron [41]. However, both DFO and DFP have been found to have little or no antimicrobial activity for *P. aeruginosa*, *K. pneumoniae*, *E. coli*, *Acinetobacter baumannii* and other nosocomial bacterial pathogens [42]. DIBI has been shown to reduce blood *A. baumannii* bacterial burdens in infected mice [43].

Finally, *Clostridia* and *Bacilli*, both Gram-positive genera, were the two other major groups found in PLF samples following CASP surgery. *Clostridia* are obligate anaerobes and include *C. difficile*, which is the most common species for hospital-acquired infection and is part of the antibiotic resistance threat [44]. It can cause much more severe sepsis if it is the causative agent. The *Clostridia* class also include *C. perfringens*. *C. perfringens* is responsible for food poisoning and can cause necrosis upon endospore release into wounds. In our findings, there was a higher distribution of *Clostridia* with DFO and DFP treatments; this change in microbiome diversity could be detrimental to health, and thus, DFO and DFP’s effects on the microbiota should be further explored. Interestingly, intravenous DIBI has been shown to eradicate their distribution [12].

*Bacilli* are aerobes and facultative anaerobes. Though they are part of the healthy gut microbiome, some of their species such as *B. cereus* can induce bacteremia and peritonitis in immunocompromised patients [45]. DIBI did not change *Bacilli* diversity in CASP, though DFO and DFX appear to have increased *Bacilli* proportions. Although not detected in the PLF samples analyzed, within this class, *Staphylococcus* imposes a major health burden on healthcare systems. *Staphylococci* are a problem in surgical site infections and ventilator-associated infections, which makes them the second most common causative agents of hospital-acquired infections [46,47,48]. Vancomycin-resistant and methicillin-resistant *S. aureus* strains are extremely difficult to treat and need immediate attention. DIBI has been shown to effectively reduce *S. aureus* growth in vivo and in vitro [49,50].

One limitation of the CASP model was the continuous fecal matter leakage through the stent. Although this helped to mimic the heterogeneity in infection severity as observed in some human sepsis patients, it made it difficult to analyze the treatments’ effects in the context of infection severity. We were not able to adjust the readouts based on the amount of fecal matter leakage present. To overcome this limitation, pre-measured feces could be introduced into the peritoneal cavity as part of the fecal slurry model [51,52]. This would better ensure consistent bacterial inoculum in each mouse and allow a further improved assessment of the effects of the different chelators, especially regarding their infection microbiome alterations. Further limitations can also be identified in the animal models we have utilized. Young, healthy, male mice were used, while in sepsis of humans, age is a risk factor, with the older population being more vulnerable to developing sepsis after an infection. Additionally, human patients often suffer from co-morbidities such as hypertension, auto-immune diseases, chronic obstructive pulmonary disorder or chronic renal failure, which can be positively correlated with mortality and ICU re-admissions [53,54,55,56]. Additionally, we did not control for sex in our study. Future experiments with older male and female mice and with co-morbidities could be investigated. Though murine models are effective in assessing the effect of treatments and they are genetically and physiologically similar to humans [57], they are not a full substitute to human models. Thus, findings in rodents must be carefully interpreted for transition into human clinical trials. Another limitation is the short study time frame of these models, which did not comprehensively assess the long-term effects of iron chelation. Even though iron chelation would most likely be administered as an acute treatment, for example, over 10 days, the long-term effects of reducing systemic iron levels should be closely investigated as sepsis patients are at risk for infection progression and having an immunocompromised state. In this regard, DIBI’s relatively short plasma half-life could be beneficial as its plasma levels (and thus, degree of iron chelation) could be better controlled. Additionally, DIBI has exhibited no adverse hematological effects in 14-day chronic systemic toxicity testing with 200 mg/kg daily repeat administrations to rats [43]. Last but not least, we compared the treatment effects of the different iron chelators after adjusting for similar iron-binding capacity, but the timing after the event and the mode of administration also play an important role in the anti-inflammatory and anti-ROS activity of each drug. Usually, following the initial insult, the inflammatory response increases gradually and reaches a zenith after 10–12 h, associated with circulatory collapse. At that time, IL-6 reached the peak value. The administration of each molecule as a bolus or via continuous infusion may have different results. For instance, an early bolus followed by infusion of DFO was sufficient to completely block IL-6 and ROS generation in a sepsis-like syndrome with multiorgan dysfunction following acute hepatic ischemia [58].

## 5. Conclusions

The present study investigated the impact of therapy with various iron chelators on both the innate immune response and bacterial growth, utilizing two different models of sepsis. This was the first time that the three FDA-approved iron chelators—DFO, DFP and DFX—and the novel synthetic iron chelator DIBI were compared in experimental sepsis. In endotoxemia, DFO, DFP and DIBI effectively reduced excessive immune activation, as shown by attenuated leukocyte–endothelial interactions and restored capillary perfusion in the intestinal microcirculation measured by IVM. In experimental sepsis induced by CASP infection, we observed a reduction in leukocyte–endothelial interactions with DFO, DFP and DIBI treatment. Iron chelation therapy did not significantly impact bacterial growth, with the exception of DIBI, which significantly reduced peritoneal bacterial burdens. The chelators appeared to cause potentially important infection-related peritoneal microbiome changes that warrant further studies.

In conclusion, these results support the immunomodulatory role of iron chelation in sepsis, in particular its therapeutic potential to dampen the hyperactivated immune response. Additionally, these results suggest a promising role for DIBI as both an anti-inflammatory and an anti-bacterial treatment, including therapeutic potential for antibiotic-resistant infections. Further pharmacological and immunological investigations are warranted before clinical studies can be considered.

## Data Availability

Data are contained within the article.
